# Match Analysis in Women’s Tennis on Clay, Grass and Hard Courts

**DOI:** 10.3390/ijerph19137955

**Published:** 2022-06-29

**Authors:** Iván Prieto-Lage, Adrián Paramés-González, Juan Carlos Argibay-González, Xoana Reguera-López-de-la-Osa, Santiago Ordóñez-Álvarez, Alfonso Gutiérrez-Santiago

**Affiliations:** 1 Observational Research Group, Faculty of Education and Sport, University of Vigo, 36005 Pontevedra, Spain; ivanprieto@uvigo.es (I.P.-L.); juan.carlos.argibay@uvigo.es (J.C.A.-G.); saordonez@alumnos.uvigo.es (S.O.-Á.); ags@uvigo.es (A.G.-S.); 2 Education, Physical Activity and Health Research Group (Gies10-DE3), Galicia Sur Health Research, Institute (IIS Galicia Sur), SERGAS-UVIGO, 36208 Vigo, Spain; xreguera@uvigo.es

**Keywords:** tennis, Roland Garros, Wimbledon, US Open, Grand Slam, observational analysis

## Abstract

(1) Background: Performance indicators in tennis such as service effectiveness, rally length or final shots are key factors in determining the winner of the match, although there is little research in the female category. The purpose of this research is to understand the game model in female tennis based on the type of surface. (2) Methods: A total of 2759 points were analyzed from three Grand Slam tournaments from 2019 on three different surfaces. We used observational methodology. (3) Results: The effectiveness of the first service was 62.4% on clay, 64.2% on grass and 67.5% on hard court. With the second service, effectiveness reduced in 5.5%, 11.2% and 14.5% from the first service, respectively. The service direction determines the efficiency and duration of the rally. The highest efficiency occurs with first serves to the T zone or wide zone (regardless of the service side) in short rallies (from 64.9% on clay to 86.3% on hard court). Serving to the centre reduces the chances of success (between 53.1% and 69.9%) and increases the rally length. Between 64.8% (clay) and 75.9% (hard court) of points played on first serve ended in a short rally, while on second serve it was 56.2% (clay) to 61.7% (grass). (4) Conclusions: The data of the effectiveness of the sequences of specific plays can help in the trainings of professional female tennis players.

## 1. Introduction

Tennis is a sport that encompasses millions of players and fans all around the world [1]. As a sport, it is characterized by intermittent dynamics, with moderate and high intensity interval efforts, caused by repetitive actions of short duration and with great intensity [2]. Professional tennis performance indicators are increasingly attracting the attention of researchers, as they make it possible to identify the reasons for winning or losing a match [3,4,5].

One of the main performance factors is the type of court, which influences the play style according to two different parameters: the friction coefficient and the restitution coefficient [6,7]. Fast surfaces (grass and hard court) lead to an aggressive and not very defensive style of play, while slow surfaces (clay) allow a more defensive style of play [6,8] In addition, depending on the speed of the court, the length of the rally increases, so that the playing tactics also vary [4,9].

Another performance factor to consider is the range of the rally length. There are various studies that used different ranges [9,10], although the most common is to establish three different categories for the analysis (0–4, 5–8, 9+ shots) [6]. Short rallies are usually won by the serving player, whereas when the rallies are medium or long, the probability of success is balanced between the two players [11]. The rally length and effectiveness are statistically related to the start of the point (first or second serve). Studies have reported higher effectiveness with first serve and short rally on either surface [8,9].

More performance factors include the first serve and return ratings because the match winners have the best values for these variables, regardless of sex [5,12]. Regarding the service and the effectiveness in the final point resolution, the direction is also important, which varies according to the type of court, with differences between the first and second service [13,14], varying the percentages of effectiveness.

Other performance factors are unforced errors, winners and break point percentages. Studies indicate that the proportion of unforced errors of losers was higher than that of winners [6,15]. On the other hand, there is a higher number of winners using forehand than when using backhand [5]. In addition, the player who wins the match has a better success rate in breakpoint situations [5,16].

The preceding studies show that there is little research related to performance factors in women’s tennis [9,16,17]. It is therefore important to continue to develop work in this area, as professional male and female tennis players have technical and tactical differences [14,18]. In addition, there is no research that reveals the relationship between different match sequences and effectiveness according to the surface. Therefore, the aim of this research was to find out the pattern of play in women’s tennis on clay, grass and hard court in Grand Slam tournaments and to determine the effectiveness.

## 2. Materials and Methods

### 2.1. Design

We used observational methodology [19] to detect the patterns of the tennis match.

The observational design used was nomothetic (all points played in the final rounds of top-level tournaments in 2019), follow-up (one season), and one-dimensional (there is no record of the concurrence of behaviors) [20]. A series of decisions about the sample, the observation and recording instruments and the analysis procedure are derived from this design.

### 2.2. Sample

A total of 2759 points were analyzed (964 in Roland Garros, 854 in Wimbledon and 941 in the US Open). All the points played since the quarterfinals (seven matches per tournament) from three of the four Grand Slam tournaments of the 2019 season were selected. We have selected one Grand Slam tournament on each type of surface (clay, grass and hard court, respectively). The Australian Open (hard court) has been excluded because it is played on the same type of surface as the US Open. The US Open has been selected because it is played during the same part of the season as Roland Garros and Wimbledon (June–September). The Australian Open is played in January.

The study was approved by the Ethics Committee of the Faculty of Education and Sport Science (University of Vigo, Application 02/0320).

### 2.3. Instruments

The instrument of observation used for this study is OBSTENNIS-v2, a category system that considers the different game possibilities in tennis in its criteria. It is an adaptation of OBSTENNIS [10].

**Figure 1:**
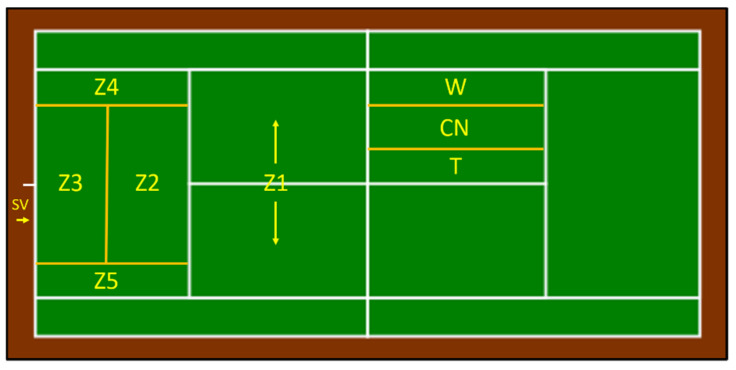
Court zones.

OBSTENNIS-v2 (Table 1 and Figure 1) consists of eight criteria that configure a category system that meets the conditions of the completeness and mutual exclusivity.

The data was recorded with LINCE v.1.4 software (Lleida, Spain) [21].

### 2.4. Procedure

The data collection was carried out by recording the matches through television channels that had the broadcasting rights of these tournaments.

After adequate training in the use of the recording instrument and the observational instrument, rigor in the recording process was guaranteed [22] by controlling the quality of the data that was going to be recorded with two expert observers in tennis through the calculation of the intra- and inter- observer’s agreements on one-tenth of the points (*n* = 275; not belonging to the final sample) and by using Cohen’s Kappa coefficient [23] calculated through LINCE software. The intra-observer agreement obtained a kappa value of 0.97 from Observer 1 and 0.98 from Observer 2. The inter-observer agreement achieved a kappa value of 0.97. Afterwards, the data was recorded by Observer 2 with the LINCE v.1.4 program.

After recording the data, we obtained an Excel spreadsheet with the sequentiality of the behaviours. The versatility of this document allowed us to make successive transformations for the different analyses [10].

### 2.5. Data Analysis

All statistical analyses were performed using the IBM-Statistical Package for the Social Sciences, version 25.0 (IBM-SPSS Inc., Chicago, IL, USA). A descriptive analysis of the study variables has been developed globally and stratified by type of service (first or second service) and side of service (deuce or advantage court). The χ2 test has been used to contrast the differences between the categories of each criterion used (intra-criterion analysis), as well as to compare the differences between the surfaces of clay, grass and hard court (inter-criterion analysis), determining the intensity of the categorical relationships through the contingency coefficient. Statistical significance was assumed for *p* ≤ 0.05.

## 3. Results

### 3.1. Traditional Statistical Analysis

**Figure 2:**
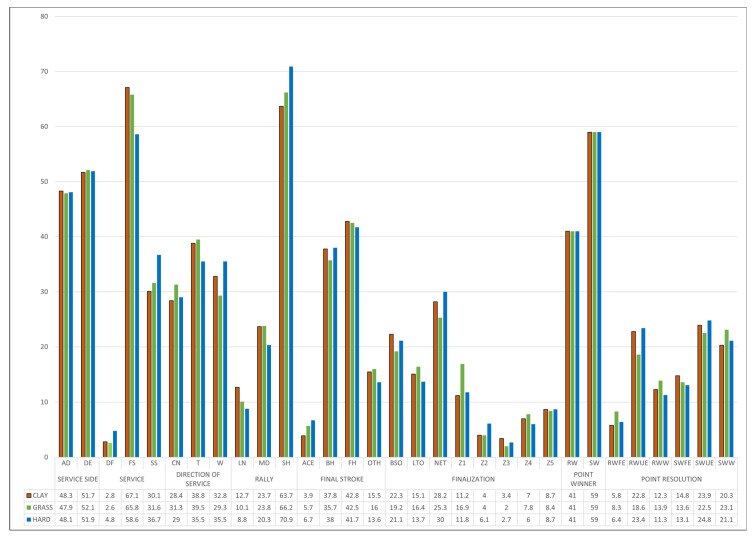
Distribution of the points played on the different surfaces in the 2019 season.

Supplementary Material Table S1 and Figure 2 present the descriptive analysis of the study that was observed, differentiating it by playing surface.

Except in the criteria side of service, statistically significant differences (*p* ≤ 0.05) were observed in the categories of each of the criteria of the three surfaces analyzed (χ2 intra-criterion). On the other hand, there were significant differences (*p* ≤ 0.05), in the comparative analysis of the surfaces (χ2 inter-criterion), in the service criteria (greater number of first services as the surface is slower), rally length (greater number of medium and long rallies on clay) and point resolution. In general, points with first service (58.6–67.1%) and directed to the T zone (35.5–38.8%) were more frequent. Short rallies predominated (63.7–70.9%), a final shot with a forehand stroke (41.7–42.8%), the serving player winning (59%) after an unforced error by the receiver (22.5–24.8%).

**Figure 3:**
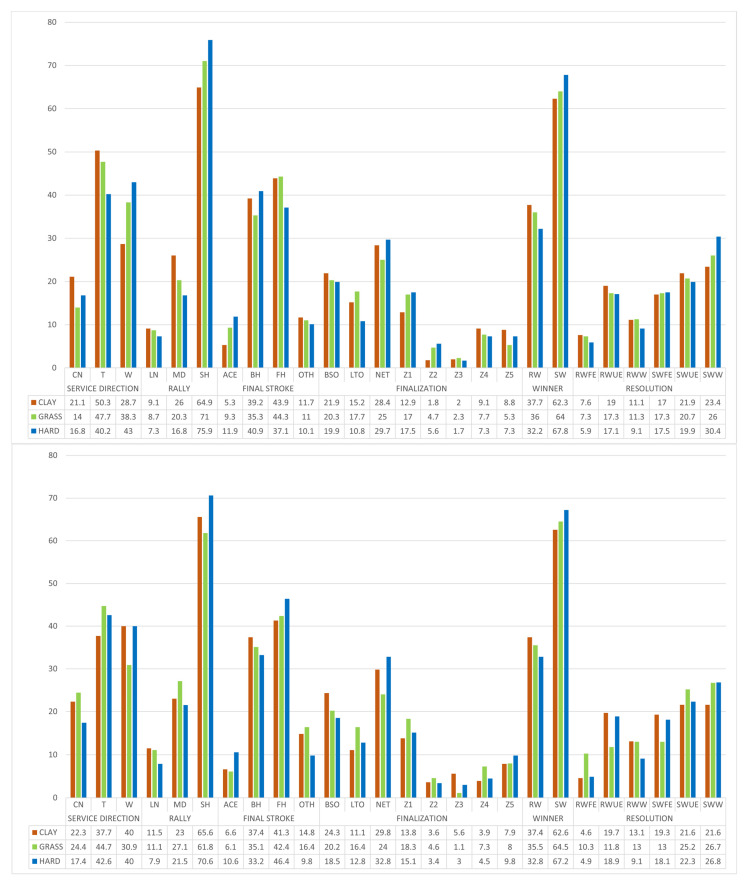
Distribution of the points played with first service to the deuce and the advantage side.

**Figure 4:**
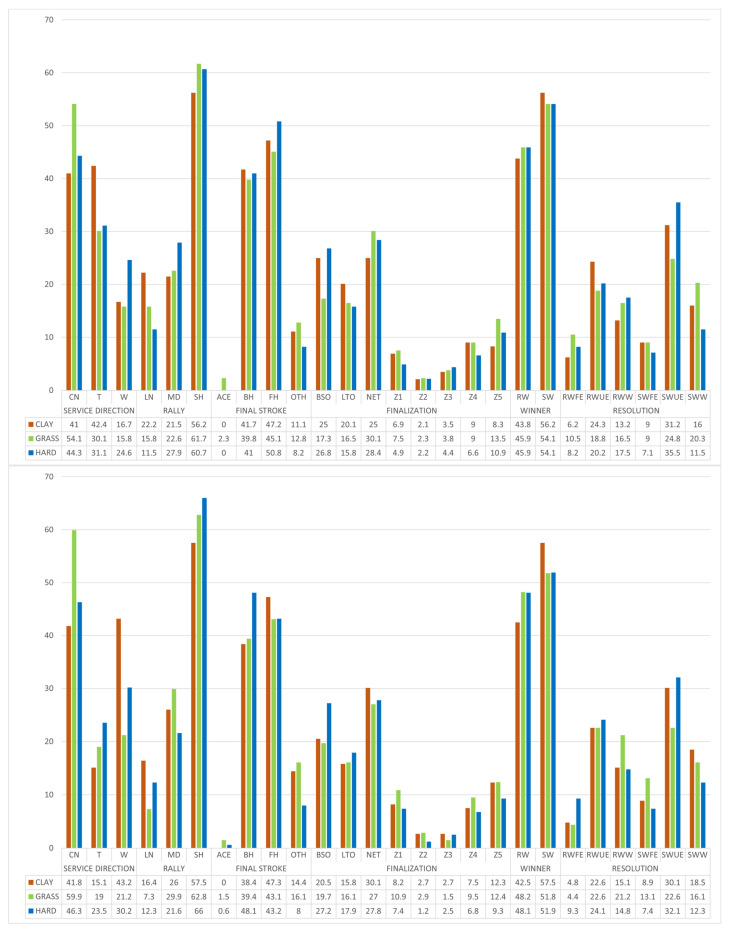
Distribution of the points played with second service to the deuce and the advantage side.

In Supplementary Material Tables S2 and S3, Figure 3 and Figure 4, the same previous analysis was done, but differentiating by service (first or second) and place of service (deuce or advantage court).

The χ2 intra-criterion analysis indicated that there were significant differences (*p* ≤ 0.05) in all the criteria of the three surfaces when studied independently, both for the first and second service and on both sides. When comparatively analyzing the data between the three surfaces, significant differences were observed (*p* ≤ 0.05). With the first service from the deuce court, the differences were found in the service direction (on clay, fewer services to the wide zone and more serves to the center zone) and in the rally length (there are more medium or long rallies when the surface is slower). With the first service from the advantage side, the differences were evidenced in the point resolution (greater number of errors hitting the ball out of bounds on clay and more shots to the net on the hard-court) and point resolution (it changes on grass with respect to the two other surfaces). With second service, significant differences were observed only in the service direction (both on the deuce and advantage courts), tending to the T zone on clay and to the center on grass and hard court.

### 3.2. Sequential Analysis

Supplementary Material Table S4 presents an analysis of the effectiveness by patterns depending on the service side and surface type.

#### 3.2.1. Sequential Analysis of FIRST Service

**Figure 5:**
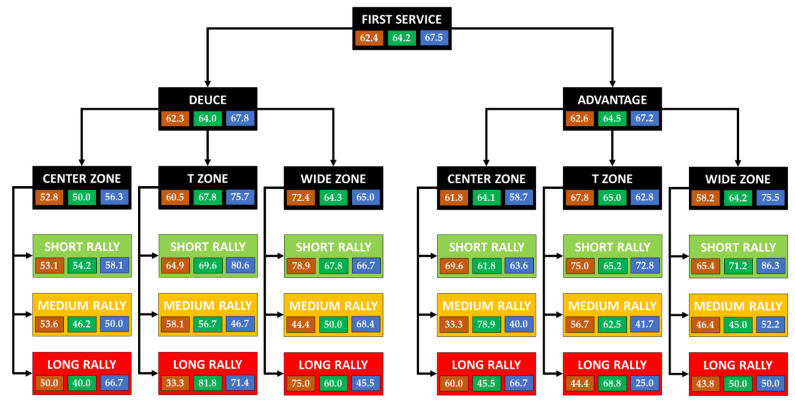
Efficiency analysis with the first service.

Figure 5 shows an analysis of the efficiency with the first service.

The tennis players were 62.4% efficient on clay, 64.2% on grass and 67.5% on hard court. A similar performance was appreciated on the three surfaces from the deuce and advantage courts, although there were differences between them. On the three surfaces (without differentiation by service side), there was a higher performance in the short rallies (69–66.9–73.8%, respectively). In the medium and long rallies, the effectiveness ranged between 47–61.8%.

Without considering the rally, in points with a service to the center, there was greater effectiveness from the advantage court (range 58.7–64.1%) than from the deuce court (range 50–56.3%) on all three surfaces. While serving to the T zone, there was a similar performance from both sides on clay and grass (range 60.5–67.8%). On hard court, the difference was notable between the deuce-advantage courts (75.7–62.8%). While serving to the wide zone, about 64% of the points were won from both sides on grass, while the effectiveness on clay was much higher on the deuce court (72.4–58.2%). On hard court, just the opposite happened (65–75.5%).

In short rallies, the efficiency was similar on clay and grass from both sides (68–70%; 67.1–66.7%, respectively), while on hard court, the difference was greater (71.4–76.5%). On the three surfaces, 55% of the points were won from the deuce court and service to the center, while those directed to the center from the advantage court were around 65%. With service to the T zone, the effectiveness ranged between 65–75% on clay and grass from both sides, while on hard court, it was 80.6–72.8%. At the wide zone points, the efficiency was 78.9–65.4% on clay and 67.8–71.2% on grass. On hard court, there was a great difference between a service from deuce-advantage court (66.7–86.3%). Regardless of the surface, although it was somewhat more remarkable on grass, the points won with service to the center were produced after an unforced error by the opponent. If the service was to the T wide zone, there was a large reduction in the points won with unforced error, and the winners and forced errors increased, especially on grass and hard court.

In medium-length rallies on the clay court, a higher efficiency was observed from the deuce court (53.9–48.6%). The same happened on hard court (56.3–45.6%). On the contrary, on grass, the yield was reversed (52.5–62%). The effectiveness, in general terms, was lower compared to the short rallies. The services directed to the center from the deuce court had an efficiency of approximately 50% on all surfaces, while from the advantage court, it did not exceed 40% on clay and hard court, but much higher on grass (78.9%). In the services directed towards the T zone, the efficiency was very similar on clay and grass (58.1–56.7% y 56.7–62.5%, respectively) and lower on hard court (46.7–41.7%). With services to the wide zone, the performance was approximately 45% on clay and grass from both sides, while on hard court, there were higher values (68.4–52.2%). On all three surfaces, most of the points started and won with service to the center were produced after an unforced error by the opponent. With service to the T or wide zone, unforced errors continued to stand out, but there was a considerable increase of forced errors on clay and grass and from winners on hard courts.

In the long rally points, the efficiency was 45.2–48.6% on clay, 65.4–58.6% on grass and 57.1–42.9% on hard courts, with a clear difference among them. With service to the center, the efficiency ranged between 40–45% on clay and grass from both sides, except from the advantage court on clay (60%). On hard court, it was 66.7% from both sides. The service to the T zone obtained an approximate yield of 40% from both sides on clay, while it was higher from the deuce court (81.8–68.8%) on grass. On hard court, there was a large difference between the sides of service (71.4–25%). With serves to the wide zone, a higher yield was obtained from the deuce court on clay (75–43.8%) and grass (60–50%). On a hard court, the efficiency was around 50% from both sides. Among the points won, the points that were resolved with a winner after serving to the T zone on grass playing from the advantage court (7/11 points; 63.6%), or winners from the deuce side on hard court (4/5 points; 80%). In any case, the sample indicated these types of points has been low.

#### 3.2.2. Sequential Analysis of Second Service

**Figure 6:**
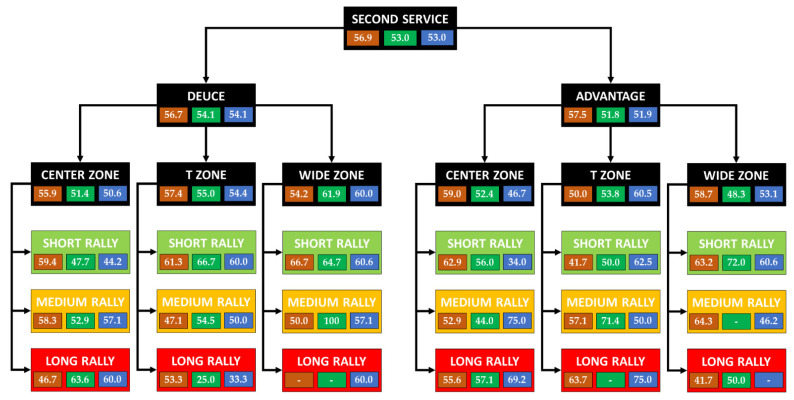
Efficiency analysis with the second service.

Figure 6 shows an analysis of the efficiency with the second service.

With second service, the efficiency was 56.9% on clay and 53% on grass and hard court (decreasing by 5.5%, 11.2% and 14.5% in relation to the first serve, respectively). A similar performance was observed from the deuce and advantage court with 56.7–57.5% on clay, 54.1–51.8% on grass and 54.1–51.9% on hard court.

The efficiency as a function of the rally (short, medium and long) on clay was 60.6–55.1–48.2%. It was 57.1–46.5–45.2% on grass and 51.4–55.8–56.1% on hard court.

In points that start with a service to the center (without considering the rally length), the efficiency was 55.9–59.0% on clay, 51.4–52.4% on grass and 50.6–46.7% on hard court. In the points with a service directed to the T zone, an efficiency of approximately 55% was evidenced on three surfaces from both sides, except for the hard court, where from the advantage court, it amounted to 60.5%. With a serve to the wide zone, over 55% of the points were won from both sides on clay, while there was a higher performance from the deuce side (61.9–48.3%) on grass. The same happened on hard court (60–53.1%).

In short rallies on clay, service efficiency was similar from both sides (61.7–59.5%). Both on grass (42.9–58.1%) and on hard court (54.1–48.6%), there was a greater difference in the effectiveness depending on the serving side. The serve to the center obtained a performance of 60% from both sides on clay, while on grass it was 47.7–56% and on hard court, it was 44.2–34%. The service performance to the T zone was around 60% on clay and hard courts, with 66.7% on grass from the deuce court. It was 41.7–50.0–62.5% from the advantage court. Among the points won, those that occurred after an unforced error by the opponent stood out, especially on grass, after a service from the advantage court to the center (18/28 points; 64.3%), or on hard court from the deuce court after serving to the wide zone (17/30 points; 85%). On clay, the winners from the deuce court with a serve to the center stood out (in the points won) (7/19 points; 36.8%). Regarding forced errors, the highest value occurred on grass (5/13 points; 38.5%) after serving from the advantage court to the T zone.

In medium-length rallies, the efficiency ranged between 50–60% on the three surfaces from both sides, except that the points started from the advantage court on grass, where it was 39%. In the points with service to the center zone, they won approximately 50% on all three surfaces, except for the advantage court on grass (44%) and hard court (75%). In services to the T and wide zone, the efficiency was approximately 50% on the three surfaces, except on grass, where the greatest efficiency was from the advantage court and service to the T zone (71.1%). In this type of rally, unforced errors on all three surfaces stand out. In points won after serving from the deuce court to the center, on hard court, there are a high number of winners compared to the rest of the surfaces (7/16 points; 43.8%). Also, on grass, after serving to the center from the advantage court, there were several forced errors afterwards (6/11 points; 54.5%).

In long rallies, efficiency was around 50% on all three surfaces from both sides, except for points from the advantage side on hard court (60%). Of the points with service to the center on clay, 46.7–55.6% won, 63.6–57.1% on grass and 60.0–69.2% on hard court from both sides. With services to the T zone, the efficiency was 61.5–66.7% on clay, while on the other surfaces there was an inconclusive effectiveness due to the low number of points recorded. Something similar happened in service to wide zone, no conclusions can be drawn from the data due to its small sample. Regarding the way of earning the points, they were mainly produced by unforced errors. We highlight the winners on clay after a service to the wide zone from the advantage side (80%).

## 4. Discussion

### 4.1. Performance Indicators

This research aims to expand the scarce information on the effectiveness of the different women’s game patterns depending on the surface (clay, grass or hard court) in the final rounds of Grand Slam.

The results obtained on the effectiveness of the first service are below those found in other investigations [9,24], where they observed 69% on clay, 74% on grass and 71% on hard court (in our study, 62.4–64.2–67.5%, respectively). Regarding the effectiveness of the second service, it is in agreement, except on clay, with what was found by previous research [9,24], since they observed 51% on clay, 52% on grass and 50% on hard court (56.9–53–53% in our case).

If we analyze the zone to which the service is directed, it has been shown that, on hard court with first services, 44.8–39.9% (deuce-advantage) were directed to the T zone, 41.2–44.9% to the wide zone and 13.9–15.2% to the center. Some previous research has also analyzed the service direction, but without taking into account the side of the service [13] obtaining that, with first services on hard court, 47% go to the T zone, 36% wide zone and 16% to the center, while 41% go to the T zone with second services, 35% to the center and 23% wide zone. On clay, there are also investigations that studied the service direction [25], concluding that, with first service, 38% go to the wide zone, 34% to the center and 27% to the T zone. With second services, it was evidenced that 31% go to the wide zone, 57% to the center and 21% to the T zone. In our case, also on clay (distinguishing by service side), 48.8–37.2% of the first services were to the wide zone, 17.8–22% to the center and 33.3–40.8% to the T zone. With second services, 16–44% was to the wide zone, 40.7–42.9% to the center and 43.2–13.1% to the T zone.

Regarding the number of points played depending on the type of rally, the results obtained coincide with those found in other investigations both on clay and on grass [5,6]. Thus, on clay and grass, respectively, 65.1–65.9% of the points were played with short rallies, 23.4–24.1% with medium rallies, and 11.5–10% with long rallies (in our case, it was 65.2–66.7% -short rally-, 24.6–23.5% -medium rally- and 10.2–9.8% -long rally-).

If we focus on the final stroke and the way to win the point, the results obtained agree with those found in other studies [6,25,26], since the server wins a greater number of points due to unforced errors by the opponent on the three surfaces, followed by winners. In addition, there are more frequent winners with a forehand stroke than with a backhand stroke to finish the points [5].

### 4.2. Play Sequences

There is not that much research that analyzes the play sequences and their effectiveness in tennis. In an investigation from more than a decade ago [9], an analysis was carried out based on service, rally length (1–2 shots, 3–4 shots and 5+) and surface (clay, grass and hard). With the first serve, they showed high effectiveness in rallies of 1–2 shots (88% on clay, 95% on grass and 91% on hard). With 3–4 rally shots, it decreased to 55–54–51%. In points with 5+ shots, the effectiveness was 53–50–48%. With second serve, effectiveness in rallies of 1–2 shots was 42–43–49%. With 3–4 shots, it was 46–41–43%, and with rallies of 5+, it was 47–43–51%.

Although it is difficult to make a clear comparison between studies, due to the established rally ranges, we can affirm that there has been a trend change over the years. Currently, the grass surface is not where there is greater effectiveness in short rallies. According to the data analyzed, with first serve, the greatest performance is on hard court, followed by clay. In long rallies with first service, the highest effectiveness was obtained on grass, followed by the hard court. With second service, once again, there is greater effectiveness in short rallies on clay, followed by grass and finally hard court. In long rallies, the highest performance was on hard court, followed by clay and grass. In any case, the study shows that the trend continues: on fast surfaces (hard court and grass), there are more points with a short rally length; on slow surfaces (clay), there are more points with a long rally length [27,28].

In men’s tennis, there is research similar to ours, with the same rally ranges [24]. With first service, on clay, they obtained an effectiveness of 46.4–64.9–55.2% depending on the rally. On grass, 75.6–53.8–46.2% and on hard court, 73.4–53.4–63.9%. All these values are very different from those observed in the female category. The very effective serve-volley strategy on fast surfaces that was pointed out as an explanation of the performance in short rallies in men’s tennis [3], is not evidenced in women’s tennis. This confirms the need for further research on the performance variables of women’s tennis.

### 4.3. Practical Recommendations

With the data from this research, tennis coaches and players should consider the following aspects:

Only 10% of the points ended with a long rally (9+ shots). On all three surfaces, most points ended with less than five shots (63.7–66.2–70.9%). Training sessions should consider these data for work-pause ranges, considering that the pause between points is 25 s per regulation.

On all three surfaces the probability of winning/losing the point by serving is 59.0–41.0%. This means that a service break is quite probable and the chance of recovering the lost serve is high. This is an important statistic for psychological training.

As is well known, the first serve is more effective than the second serve. In any case, the service direction has been determinant in the final efficiency of the point. If we contemplate short rallies with a first serve (the tennis player’s objective when serving—it is also the most frequent sequence), serving to the center zone decreases the probability of success on both sides of the serve. If serving to the deuce court, it is more effective to serve to the T zone on fast surfaces (grass and hard court) and to the wide zone on clay. If serving to the advantage side, it is more effective to serve to the wide zone on fast surfaces (grass and hard court) and to the T zone on clay courts. In any case, it is recommended to use Figure 5 and Figure 6 for a more extensive analysis by type of service, surface and rally. Coaches should consider the efficiency of these service patterns when training this technical element.

Data do not indicate many differences in the point resolution (winners, unforced errors and forced errors) depending on the surface. There is a very similar probability that the point will end with a winner or forced error (approximately 55%) or an unforced error (45%). Therefore, a defensive game (waiting for the opponent’s error) does not seem to be effective even on clay. The trend in women’s tennis indicates that points are becoming shorter and shorter and that players should look for winners in the outer areas of the court (zone 4 and zone 5). Tennis players should consider this information in their tactical game training.

### 4.4. Limitations

Only Grand Slam matches have been considered since the quarterfinals, without considering the previous rounds. Despite the volume of points analyzed, it is possible that the results could vary if all the matches of the tournament were analyzed. The analysis of the game situations has not considered whether the tennis player was left-handed or right-handed. The speed of the service has not been considered, an element that could be relevant in the performance of the point

## 5. Conclusions

The first serve is essential for the effectiveness on all types of rallies and surface, although the shorter the rally and the faster the court, the more effective it is. Serving with a second serve lowers the chances of winning the point. The zone where the service is directed to is also important, especially when using the first serve. Service to the T or wide zone increases the chances of success. Serving to the center zone reduces effectiveness and generates a longer rally. In the point resolution, unforced errors are those that are repeated the most on the three surfaces, followed by winners, being on hard court, where they occur the most.

## Figures and Tables

**Table 1:** Observation instrument OBSTENNIS-v2.

Criteria	Code	Description
SURFACE	CL	Clay (Roland Garros’ match).
	GR	Grass (Wimbledon’ match).
	HC	Hard court (US Open match).
SERVICE SIDE	AD	The service is done to the advantage side.
	DE	The service is done to the deuce side.
SERVICE	DF	Double fault.
	FS	Point is played with first service.
	SS	Point is played with second service.
SERVICE DIRECTION	CN	Service to the center of the service quadrant.
	T	Service to the T zone.
	W	Service to the wide zone.
RALLY LENGTH	LN	Long rally (9+ shots). Does not count the service.
	MD	Medium rally (5–8 shots). Does not count the service.
	SH	Short rally (0–4 shots). Does not count the service.
FINAL STROKE	ACE	Direct service.
	BH	Backhand stroke.
	FH	Forehand stroke.
	OTH	Other type of stroke (drop shot, smash, volley…).
POINT FINALIZATION	Z1-Z5	Court zone where ball finally lands (only for winning strokes).
	NET	Final stroke goes to the net.
	LTO	Final stroke goes out of bounds on the side.
	BSO	Final stroke goes out of bounds at the base line.
WINNER OF THE POINT	RW	The player that receives wins the point.
	SW	The player that serves wins the point.
POINT RESOLUTION	RWFE	The receiver wins with a forced error from the opponent.
	RWUE	The receiver wins without a forced error from the opponent.
	RWW	The receiver wins with a winning stroke.
	SWFE	The player that serves wins with a forced error from the opponent.
	SWUE	The player that serves wins without a forced error from the opponent.
	SWW	The player that serves wins with a winning stroke.

## Data Availability

Not applicable.

## Supplementary Materials

The following supporting information can be downloaded at: https://www.mdpi.com/article/10.3390/ijerph19137955/s1.
