# Supplementary material for: Match Analysis in Women’s Tennis on Clay, Grass and Hard Courts
Source: Int J Environ Res Public Health. 2022 Jun 29;19(13):7955. doi: 10.3390/ijerph19137955 (PMC9266198; doi:10.3390/ijerph19137955)
Supplement: Supplementary file 1 [file ijerph-19-07955-s001.zip › ijerph-1740673-supplementary.pdf]

Table S1. Distribution of the points played on the different surfaces in the 2019 season, analysis of the distribution of the categories of each by surface (χ2 intra-criteria) and comparative analysis among surfaces (χ2 inter-criteria).

Criteria	Code	Clay		χ2 Intra Criteria CL	Grass		χ2 Intra Criteria GR	Hard court		χ2 Intra Criteria HC	χ2 Inter criteria	Coef. Contig.
		n	%		n	%		n	%			
SERVICE SIDE	AD	466	48.3%	χ2=1.062	409	47.9%	χ2=1.518	453	48.1%	χ2=1.302	χ2=(2,N=2759)= .036;	.004
	DE	498	51.7%	*p*= .303	445	52.1%	*p*=.218	488	51.9%	*p*=.254	*p*=.982	
SERVICE	DF	27	2.8%	χ2=602.716	22	2.6%	χ2=513.311	45	4.8%	χ2=412.829	χ2=(4,N=2759)=	.087
	FS	647	67.1%	*p*<.000	562	65.8%	*p*<.000	551	58.6%	*p*<.000	21.069;	
	SS	290	30.1%		270	31.6%		345	36.7%		*p*<.000	
SERVICE DIRECTION	CN	274	28.4%	χ2=15.693	267	31.3%	χ2=14.939	273	29.0%	χ2=9.909	χ2=(4,N=2759)=	.057
	T	374	38.8%	*p*<.000	337	39.5%	*p*<.001	334	35.5%	*p*<.019	8.870;	
	W	316	32.8%		250	29.3%		334	35.5%		*p*=.064	
RALLY	LN	122	12.7%	χ2=417.320	86	10.1%	χ2=438.143	83	8.8%	χ2=615.617	χ2=(4,N=2759)=	.071
	MD	228	23.7%	*p*<.000	203	23.8%	*p*<.000	191	20.3%	*p*<.000	13.890; *p*=.008	
	SH	614	63.7%		565	66.2%		667	70.9%			
FINAL STROKE	ACE	38	3.9%		49	5.7%		63	6.7%			
	BH	364	37.8%	χ2=391.643	305	35.7%	χ2=298.056	358	38.0%	χ2=343.510	χ2=(6,N=2759)=	.059
	FH	413	42.8%	*p*<.000	363	42.5%	*p*<.000	392	41.7%	*p*<.000	9.755; *p*=.135	
	OTH	149	15.5%		137	16.0%		128	13.6%			
FINALIZATION	BSO	215	22.3%		164	19.2%		199	21.1%			
	LTO	146	15.1%		140	16.4%		129	13.7%			
	NET	272	28.2%		216	25.3%		282	30.0%			
	Z1	108	11.2%	χ2=424.747	144	16.9%	χ2=317.016	111	11.8%	χ2=434.736	χ2=(14,N=2759)=	.107
	Z2	39	4.0%	*p*<.000	34	4.0%	*p*<.000	57	6.1%	*p*<.000	32.172; *p*=.004	
	Z3	33	3.4%		17	2.0%		25	2.7%			
	Z4	67	7.0%		67	7.8%		56	6.0%			
	Z5	84	8.7%		72	8.4%		82	8.7%			
WINNER	RW	395	41.0%	χ2=31.407	350	41.0%	χ2=27.770	386	41.0%	χ2=30.352	χ2=(2,N=2759)= .000;	.000
	SW	569	59.0%	*p*<.000	504	59.0%	*p*<.000	555	59.0%	*p*<.000	*p*= 1.000	
RESOLUTION	RWFE	56	5.8%		71	8.3%		60	6.4%			
	RWUE	220	22.8%		159	18.6%		220	23.4%			
	RWW	119	12.3%	χ2=140.535	119	13.9%	χ2=84.726	106	11.3%	χ2=157.332	χ2=(10,N=2759)=	.077
	SWFE	143	14.8%	*p*<.000	116	13.6%	*p*<.000	123	13.1%	*p*<.000	16.387; *p*=.089	
	SWUE	230	23.9%		192	22.5%		233	24.8%			
	SWW	196	20.3%		197	23.1%		199	21.1%			

Table S2. Distribution of the points played with first service to the deuce and the advantage side, distribution analysis of the categories of each criteria by surface (χ2 intra-criteria) and comparative analysis among surfaces (χ2 inter-criteria).

Criteria	Code	Clay		χ2 Intra Criteria CL	Grass		χ2 Intra Criteria GR	Hard court		χ2 Intra Criteria HC	χ2 Inter criteria	Coef. Contig.
		n	%		n	%		n	%			
**FIRST SERVICE (DEUCE)**												
SERVICE DIRECTION	CN	72	21.1%	χ2=47.228 *p*<.000	42	14.0%	χ2=54.380 *p*<.000	48	16.8%	χ2=35.587 *p*<.000	χ2=(4,N=928)= 17.662; *p*=.001	.137
	T	172	50.3%		143	47.7%		115	40.2%			
	W	98	28.7%		115	38.3%		123	43.0%			
RALLY	LN	31	9.1%	χ2=168.228 *p*<.000	26	8.7%	χ2=197.660 *p*<.000	21	7.3%	χ2=236.734 *p*<.000	χ2=(4,N=928)= 9.725; *p*=.045	.102
	MD	89	26.0%		61	20.3%		48	16.8%			
	SH	222	64.9%		213	71.0%		217	75.9%			
FINAL STROKE	ACE	18	5.3%	χ2=153.673 *p*<.000	28	9.3%	χ2=110.640 *p*<.000	34	11.9%	χ2=90.531 *p*<.000	χ2=(6,N=928)= 12.122; *p*=.059	.114
	BH	134	39.2%		106	35.3%		117	40.9%			
	FH	150	43.9%		133	44.3%		106	37.1%			
	OTH	40	11.7%		33	11.0%		29	10.1%			
FINALIZATION	BSO	75	21.9%	χ2=163.731 *p*<.000	61	20.3%	χ2=120.960 *p*<.000	57	19.9%	χ2=136.322 *p*<.000	χ2=(14,N=928)= 19.206; *p*=.157	.142
	LTO	52	15.2%		53	17.7%		31	10.8%			
	NET	97	28.4%		75	25.0%		85	29.7%			
	Z1	44	12.9%		51	17.0%		50	17.5%			
	Z2	6	1.8%		14	4.7%		16	5.6%			
	Z3	7	2.0%		7	2.3%		5	1.7%			
	Z4	31	9.1%		23	7.7%		21	7.3%			
	Z5	30	8.8%		16	5.3%		21	7.3%			
WINNER	RW	129	37.7%	χ2=20.632 *p*<.000	108	36.0%	χ2=23.520 *p*<.000	92	32.2%	χ2=36.378 *p*<.000	χ2=(2,N=928)= 2.156; *p*=.340	.048
	SW	213	62.3%		192	64.0%		194	67.8%			
RESOLUTION	RWFE	26	7.6%	χ2=39.298 *p*<.000	22	7.3%	χ2=39.520 *p*<.000	17	5.9%	χ2=64.014 *p*<.000	χ2=(10,N=928)= 5.192; *p*=.878	.075
	RWUE	65	19.0%		52	17.3%		49	17.1%			
	RWW	38	11.1%		34	11.3%		26	9.1%			
	SWFE	58	17.0%		52	17.3%		50	17.5%			
	SWUE	75	21.9%		62	20.7%		57	19.9%			
	SWW	80	23.4%		78	26.0%		87	30.4%			
**FIRST SERVICE (ADVANTAGE)**												
SERVICE DIRECTION	CN	68	22.3%	χ2=16.964 *p*<.000	64	24.4%	χ2=16.771 *p*<.000	46	17.4%	χ2=30.709 *p*<.000	χ2=(4,N=832)= 9.020; *p*=.061	.104
	T	115	37.7%		117	44.7%		113	42.6%			
	W	122	40.0%		81	30.9%		106	40.0%			
RALLY	LN	35	11.5%	χ2=148.689 *p*<.000	29	11.1%	χ2=105.855 *p*<.000	21	7.9%	χ2=172.649 *p*<.000	χ2=(4,N=832)= 5.432; *p*=.246	.081
	MD	70	23.0%		71	27.1%		57	21.5%			
	SH	200	65.6%		162	61.8%		187	70.6%			
FINAL STROKE	ACE	20	6.6%	χ2=105.452 *p*<.000	16	6.1%	χ2=87.466 *p*<.000	28	10.6%	χ2=102.291 *p*<.000	χ2=(6,N=832)= 10.419; *p*=.108	.111
	BH	114	37.4%		92	35.1%		88	33.2%			
	FH	126	41.3%		111	42.4%		123	46.4%			
	OTH	45	14.8%		43	16.4%		26	9.8%			
FINALIZATION	BSO	74	24.3%	χ2=162.069 *p*<.000	53	20.2%	χ2=100.931 *p*<.000	49	18.5%	χ2=148.313 *p*<.000	χ2=(14,N=832)= 23.926; *p*=.047	.167
	LTO	34	11.1%		43	16.4%		34	12.8%			
	NET	91	29.8%		63	24.0%		87	32.8%			
	Z1	42	13.8%		48	18.3%		40	15.1%			
	Z2	11	3.6%		12	4.6%		9	3.4%			
	Z3	17	5.6%		3	1.1%		8	3.0%			
	Z4	12	3.9%		19	7.3%		12	4.5%			
	Z5	24	7.9%		21	8.0%		26	9.8%			
WINNER	RW	114	37.4%	χ2=19.439 *p*<.000	93	35.5%	χ2=22.046 *p*<.000	87	32.8%	χ2=31.249 *p*<.000	χ2=(2,N=832)= 1.287; *p*=.525	.039
	SW	191	62.6%		169	64.5%		178	67.2%			
RESOLUTION	RWFE	14	4.6%	χ2=41.013 *p*<.000	27	10.3%	χ2=41.618 *p*<.000	13	4.9%	χ2=53.589 *p*<.000	χ2=(10,N=832)= 23.576; *p*=.009	.166
	RWUE	60	19.7%		31	11.8%		50	18.9%			
	RWW	40	13.1%		34	13.0%		24	9.1%			
	SWFE	59	19.3%		34	13.0%		48	18.1%			
	SWUE	66	21.6%		66	25.2%		59	22.3%			
	SWW	66	21.6%		70	26.7%		71	26.8%			

Table S3. Distribution of points played with second service to the deuce and of the advantage court. Analysis of the distribution of the categories of each criterion by surface (χ2 intra-criteria) and comparative analysis among the surfaces (χ2 inter-criteria).

Criteria	Code	Clay		χ2 Intra Criteria CL	Grass		χ2 Intra Criteria GR	Hard court		χ2 Intra Criteria HC	χ2 Inter criteria	Coef. Contig.
		n	%		n	%		n	%			
**SECOND SERVICE (DEUCE)**												
SERVICE DIRECTION	CN	59	41.0%	χ2=18.042	72	54.1%	χ2=29.970	81	44.3%	χ2=11.016	χ2=(4,N=460)= 10.713;	.151
	T	61	42.4%	*p*<.000	40	30.1%	*p*<.000	57	31.1%	*p*<.004	*p*=.030	
	W	24	16.7%		21	15.8%		45	24.6%			
RALLY	LN	32	22.2%	χ2=34.042	21	15.8%	χ2=48.917	21	11.5%	χ2=68.852	χ2=(4,N=460)= 7.773;	.129
	MD	31	21.5%	*p*<.000	30	22.6%	*p*<.000	51	27.9%	*p*<.000	*p*=.100	
	SH	81	56.2%		82	61.7%		111	60.7%			
FINAL STROKE	ACE	0	0.0%	χ2=32.667	3	2.3%	χ2=68.714	0	0.0%	χ2=54.689	χ2=(6,N=460)= 9.628;	.143
	BH	60	41.7%	*p*<.000	53	39.8%	*p*<.000	75	41.0%	*p*<.000	*p*=.141	
	FH	68	47.2%		60	45.1%		93	50.8%			
	OTH	16	11.1%		17	12.8%		15	8.2%			
FINALIZATION	BSO	36	25.0%	χ2=71.556	23	17.3%	χ2=60.383	49	26.8%	χ2=107.754	χ2=(14,N=460)= 8.472;	.134
	LTO	29	20.1%	*p*<.000	22	16.5%	*p*<.000	29	15.8%	*p*<.000	*p*=.863	
	NET	36	25.0%		40	30.1%		52	28.4%			
	Z1	10	6.9%		10	7.5%		9	4.9%			
	Z2	3	2.1%		3	2.3%		4	2.2%			
	Z3	5	3.5%		5	3.8%		8	4.4%			
	Z4	13	9.0%		12	9.0%		12	6.6%			
	Z5	12	8.3%		18	13.5%		20	10.9%			
WINNER	RW	63	43.8%	χ2=2.250	61	45.9%	χ2=.910	84	45.9%	χ2=1.230	χ2=(2,N=460)= .182;	.020
	SW	81	56.2%	*p*=.134	72	54.1%	*p*=.340	99	54.1%	*p*=.268	p=.913	
RESOLUTION	RWFE	9	6.2%	χ2=38.917	14	10.5%	χ2=14.383	15	8.2%	χ2=61.361	χ2=(10,N=460)= 10.885;	.152
	RWUE	35	24.3%	*p*<.000	25	18.8%	*p*=.013	37	20.2%	*p*<.000	*p*=.367	
	RWW	19	13.2%		22	16.5%		32	17.5%			
	SWFE	13	9.0%		12	9.0%		13	7.1%			
	SWUE	45	31.2%		33	24.8%		65	35.5%			
	SWW	23	16.0%		27	20.3%		21	11.5%			
**SECOND SERVICE (ADVANTAGE)**												
SERVICE DIRECTION	CN	61	41.8%	χ2=21.959	82	59.9%	χ2=43.460	75	46.3%	χ2=13.370	χ2=(4,N=445)= 18.844;	.202
	T	22	15.1%	*p*<.000	26	19.0%	*p*<.000	38	23.5%	*p*<.001	*p*<.001	
	W	63	43.2%		29	21.2%		49	30.2%			
RALLY	LN	24	16.4%	χ2=40.493	10	7.3%	χ2=63.956	20	12.3%	χ2=80.111	χ2=(4,N=445)= 7.798;	.131
	MD	38	26.0%	*p*<.000	41	29.9%	*p*<.000	35	21.6%	*p*<.000	*p*<.099	
	SH	84	57.5%		86	62.8%		107	66.0%			
FINAL STROKE	ACE	0	0.0%	χ2=25.329	2	1.5%	χ2=64.022	1	0.6%	χ2=113.407	χ2=(6,N=445)= 9.105;	.142
	BH	56	38.4%	*p*<.000	54	39.4%	*p*<.000	78	48.1%	*p*<.000	*p*=.168	
	FH	69	47.3%		59	43.1%		70	43.2%			
	OTH	21	14.4%		22	16.1%		13	8.0%			
FINALIZATION	BSO	30	20.5%	χ2=72.411	27	19.7%	χ2=54.825	44	27.2%	χ2=100.321	χ2=(14,N=445)= 7.264;	.127
	LTO	23	15.8%	*p*<.000	22	16.1%	*p*<.000	29	17.9%	*p*<.000	*p*=.924	
	NET	44	30.1%		37	27.0%		45	27.8%			
	Z1	12	8.2%		15	10.9%		12	7.4%			
	Z2	4	2.7%		4	2.9%		2	1.2%			
	Z3	4	2.7%		2	1.5%		4	2.5%			
	Z4	11	7.5%		13	9.5%		11	6.8%			
	Z5	18	12.3%		17	12.4%		15	9.3%			
WINNER	RW	62	42.5%	χ2=3.315	66	48.2%	χ2=.182	78	48.1%	χ2=.222	χ2=(2,N=445)= 1.280;	.054
	SW	84	57.5%	*p*=.069	71	51.8%	p=.669	84	51.9%	*p*=.637	*p*=.527	
RESOLUTION	RWFE	7	4.8%	χ2=37.123	6	4.4%	χ2=20.971	15	9.3%	χ2=44.296	χ2=(10,N=445)= 12.958;	.168
	RWUE	33	22.6%	*p*<.000	31	22.6%	*p*<.001	39	24.1%	*p*<.000	*p*=.226	
	RWW	22	15.1%		29	21.2%		24	14.8%			
	SWFE	13	8.9%		18	13.1%		12	7.4%			
	SWUE	44	30.1%		31	22.6%		52	32.1%			
	SWW	27	18.5%		22	16.1%		20	12.3%			

Table S4. Analysis of the effectiveness of the match patterns of women depending on the service and surface.

Pattern (deuce)	Clay (n=964)			Grass (n=854)			Hard court (n=941)			Pattern (advantage)	Clay			Grass			Hard court		
	Fr.	%	Ef.	Fr.	%	Ef.	Fr.	%	Ef.		Fr.	%	Ef.	Fr.	%	Ef.	Fr.	%	Ef.
FS [a]	647	67.1	62.4	562	65.8	64.2	551	58.6	67.5	-	-	-	-	-	-	-	-	-	-
FS-SW	404	-	-	361	-	-	372	-	-	-	-	-	-	-	-	-	-	-	-
FS-DE [b]	342	52.9	-	300	53.4	-	286	51.9	-	FS-AD [b]	305	47.1	-	262	46.6	-	265	48.1	-
FS-DE-SW [c]	213	52.7	62.3	192	53.2	64.0	194	52.2	67.8	FS-AD-SW [c]	191	47.3	62.6	169	46.8	64.5	178	47.8	67.2
FS-SH-SW	291 (422)	65.2	69.0	251 (375)	66.7	66.9	298 (404)	73.3	73.8	-	-	-	-	-	-	-	-	-	-
FS-MD-SW	82 (159)	24.6	51.6	76 (132)	23.5	57.6	53 (105)	19.1	50.5	-	-	-	-	-	-	-	-	-	-
FS-LN-SW	31 (66)	10.2	47.0	34 (55)	9.8	61.8	21 (42)	7.6	50.0	-	-	-	-	-	-	-	-	-	-
FS-DE-CN-SW [c]	38 (72)	17.8	52.8	21 (42)	10.9	50.0	27 (48)	13.9	56.3	FS-AD-CN-SW	42 (68)	22.0	61.8	41 (64)	24.3	64.1	27 (46)	15.2	58.7
FS-DE-T-SW [c]	104 (172)	48.8	60.5	97 (143)	50.5	67.8	87 (115)	44.8	75.7	FS-AD-T-SW	78 (115)	40.8	67.8	76 (117)	45.0	65.0	71 (113)	39.9	62.8
FS-DE-W-SW [c]	71 (98)	33.3	72.4	74 (115)	38.5	64.3	80 (123)	41.2	65.0	FS-AD-W-SW	71 (122)	37.2	58.2	52 (81)	30.8	64.2	80 (106)	44.9	75.5
FS-DE-SH-SW [d]	151 (222)	64.9	68.0	143 (213)	71.0	67.1	155 (217)	75.9	71.4	FS-AD-SH-SW	140 (200)	65.6	70.0	108 (162)	61.8	66.7	143 (187)	70.6	76.5
FS-DE-CN-SH-SW [c]	17 (32)	11.3	53.1	13 (24)	9.1	54.2	18 (31)	11.6	58.1	FS-AD-CN-SH-SW [c]	32 (46)	22.9	69.6	21 (34)	19.4	61.8	21 (33)	14.7	63.6
FS-DE-T-SH-SW [c]	74 (114)	49.0	64.9	71 (102)	49.7	69.6	75 (93)	48.4	80.6	FS-AD-T-SH-SW [c]	57 (76)	40.7	75.0	45 (69)	41.7	65.2	59 (81)	41.3	72.8
FS-DE-W-SH-SW [c]	60 (76)	39.7	78.9	59 (87)	41.3	67.8	62 (93)	40.0	66.7	FS-AD-W-SH-SW [c]	51 (78)	36.4	65.4	42 (59)	38.9	71.2	63 (73)	44.1	86.3
FS-DE-CN-SH-SW-SWFE	2	11.8	-	1	7.7	-	1	5.6	-	FS-AD-CN-SH-SW-SWFE	9	28.1	-	1	4.8	-	6	28.6	-
FS-DE-CN-SH-SW-SWUE	10	58.8	-	11	84.6	-	12	66.7	-	FS-AD-CN-SH-SW-SWUE	19	59.4	-	14	66.7	-	10	47.6	-
FS-DE-CN-SH-SW-SWW	5	29.8	-	1	7.7	-	5	27.8	-	FS-AD-CN-SH-SW-SWW	4	12.5	-	6	28.6	-	5	23.8	-
FS-DE-T-SH-SW-SWFE	19	25.7	-	23	32.4	-	20	26.7	-	FS-AD-T-SH-SW-SWFE	20	35.1	-	9	20.0	-	11	18.6	-
FS-DE-T-SH-SW-SWUE	20	27.0	-	18	25.4	-	16	21.3	-	FS-AD-T-SH-SW-SWUE	10	17.5	-	13	28.9	-	19	32.2	-
FS-DE-T-SH-SW-SWW	35	47.3	-	30	42.3	-	39	52.0	-	FS-AD-T-SH-SW-SWW	27	47.4	-	23	51.1	-	29	49.2	-
FS-DE-W-SH-SW-SWFE	23	38.3	-	14	23.7	-	17	27.4	-	FS-AD-W-SH-SW-SWFE	18	35.3	-	10	23.8	-	19	30.2	-
FS-DE-W-SH-SW-SWUE	15	25.0	-	13	22.0	-	17	27.4	-	FS-AD-W-SH-SW-SWUE	16	31.4	-	12	28.6	-	10	15.9	-
FS-DE-W-SH-SW-SWW	22	36.7	-	32	54.2	-	28	45.2	-	FS-AD-W-SH-SW-SWW	17	33.3	-	20	47.6	-	34	54.0	-
FS-DE-MD-SW [d]	48 (89)	26.0	53.9	32 (61)	20.3	52.5	27 (48)	16.8	56.3	FS-AD-MD-SW	34 (70)	23.0	48.6	44 (71)	27.1	62.0	26 (57)	21.5	45.6
FS-DE-CN-MD-SW [c]	15 (28)	31.3	53.6	6 (13)	18.8	46.2	7 (14)	25.9	50.0	FS-AD-CN-MD-SW [c]	4 (12)	11.8	33.3	15 (19)	34.1	78.9	4 (10)	15.4	40.0
FS-DE-T-MD-SW [c]	25 (43)	52.1	58.1	17 (30)	53.1	56.7	7 (15)	25.9	46.7	FS-AD-T-MD-SW [c]	17 (30)	50.0	56.7	20 (32)	45.5	62.5	10 (24)	38.5	41.7
FS-DE-W-MD-SW [c]	8 (18)	16.7	44.4	9 (18)	28.1	50.0	13 (19)	48.1	68.4	FS-AD-W-MD-SW [c]	13 (28)	38.2	46.4	9 (20)	20.5	45.0	12 (23)	46.2	52.2
FS-DE-CN-MD-SW-SWFE	2	13.3	-	2	33.3	-	2	28.6	-	FS-AD-CN-MD-SW-SWFE	-	-	-	3	20.0	-	-	-	-
FS-DE-CN-MD-SW-SWUE	9	60.0	-	3	50.0	-	1	14.3	-	FS-AD-CN-MD-SW-SWUE	2	50.0	-	9	60.0	-	4	100.0	-
FS-DE-CN-MD-SW-SWW	4	26.7	-	1	16.7	-	4	57.1	-	FS-AD-CN-MD-SW-SWW	2	50.0	-	3	20.0	-	-	-	-
FS-DE-T-MD-SW-SWFE	8	32.0	-	5	29.4	-	2	28.6	-	FS-AD-T-MD-SW-SWFE	3	17.6	-	4	20.0	-	3	30.0	-
FS-DE-T-MD-SW-SWUE	9	36.0	-	9	52.9	-	2	28.6	-	FS-AD-T-MD-SW-SWUE	10	58.8	-	11	55.0	-	7	70.0	-
FS-DE-T-MD-SW-SWW	8	32.0	-	3	17.9	-	3	42.9	-	FS-AD-T-MD-SW-SWW	4	23.5	-	5	25.0	-	-	-	-
FS-DE-W- MD -SW-SWFE	1	12.5	-	-	-	-	5	38.5	-	FS-AD-W- MD -SW-SWFE	6	46.2	-	3	33.3	-	5	41.7	-
FS-DE-W-MD-SW-SWUE	5	62.5	-	4	44.4	-	4	30.8	-	FS-AD-W-MD-SW-SWUE	3	23.1	-	3	33.3	-	5	41.7	-
FS-DE-W-MD-SW-SWW	8	25.0	-	5	55.6	-	4	30.8	-	FS-AD-W-MD-SW-SWW	4	30.8	-	3	33.3	-	2	16.7	-
FS-DE-LN-SW d	14 (31)	9.1	45.2	17 (26)	8.7	65.4	12 (21)	7.3	57.1	FS-AD-LN-SW	17 (35)	11.5	48.6	17 (29)	11.1	58.6	9 (21)	7.9	42.9
FS-DE-CN-LN-SW [c]	6 (12)	42.9	50.0	2 (5)	11.8	40.0	2 (3)	16.7	66.7	FS-AD-CN-LN-SW [c]	6 (10)	35.3	60.0	5 (11)	29.4	45.5	2 (3)	22.2	66.7
FS-DE-T-LN-SW [c]	5 (15)	35.7	33.3	9 (11)	52.9	81.8	5 (7)	41.7	71.4	FS-AD-T-LN-SW [c]	4 (9)	23.5	44.4	11 (16)	64.7	68.8	2 (8)	22.2	25.0
FS-DE-W-LN-SW [c]	3 (4)	21.4	75.0	6 (10)	35.2	60.0	5 (11)	41.7	45.5	FS-AD-W-LN-SW [c]	7 (16)	41.2	43.8	1 (2)	5.9	50.0	5 (10)	55.6	50.0
FS-DE-CN-LN-SW-SWFE	1	16.7	-	1	50.0	-	1	50.0	-	FS-AD-CN-LN-SW-SWFE	1	16.7	-	-	-	-	2	100.0	-
FS-DE-CN-LN-SW-SWUE	4	66.7	-	-	-	-	1	50.0	-	FS-AD-CN-LN-SW-SWUE	1	16.7	-	3	60.0	-	-	-	-
FS-DE-CN-LN-SW-SWW	1	16.7	-	1	50.0	-	-	-	-	FS-AD-CN-LN-SW-SWW	4	66.7	-	2	40.0	-	-	-	-
FS-DE-T-LN-SW-SWFE	1	20.0	-	2	22.2	-	2	40.0	-	FS-AD-T-LN-SW-SWFE	2	50.0	-	3	27.3	-	-	-	-
FS-DE-T-LN-SW-SWUE	2	40.0	-	3	33.3	-	3	60.0	-	FS-AD-T-LN-SW-SWUE	1	25.0	-	1	9.1	-	1	50.0	-
FS-DE-T-LN-SW-SWW	2	40.0	-	4	44.4	-	-	-	-	FS-AD-T-LN-SW-SWW	1	25.0	-	7	63.6	-	1	50.0	-
FS-DE-W-LN-SW-SWFE	1	33.3	-	4	66.7	-	-	-	-	FS-AD-W-LN-SW-SWFE	-	-	-	-	-	-	2	40.0	-
FS-DE-W-LN-SW-SWUE	1	33.3	-	1	16.7	-	1	20.0	-	FS-AD-W-LN-SW-SWUE	4	57.1	-	-	-	-	3	60.0	-
FS-DE-W-LN-SW-SWW	1	33.3	-	1	16.7	-	4	80.0	-	FS-AD-W-LN-SW-SWW	3	42.9	-	1	100.0	-	-	-	-

Table S4. Continuation.

Pattern (deuce)	Clay (n=964)			Grass (n=854)			Hard court (n=941)			Pattern (advantage)	Clay			Grass			Hard court		
	Fr.	%	Ef.	Fr.	%	Ef.	Fr.	%	Ef.		Fr.	%	Ef.	Fr.	%	Ef.	Fr.	%	Ef.
SS [a]	290	30.1	56.9	270	31.6	53.0	345	36.7	53.0	-	-	-	-	-	-	-	-	-	-
SS-SW	165	-	-	143	-	-	183	-	-	-	-	-	-	-	-	-	-	-	-
SS-DE	144	49.7	-	133	49.3	-	183	53.0	-	SS-AD	146	50.3	-	137	50.7	-	162	47.0	-
SS-DE-SW [b]	81	49.1	56.7	72	50.3	54.1	99	54.1	54.1	SS-AD-SW	84	50.9	57.5	71	49.7	51.8	84	45.9	51.9
SS-SH-SW	100 (165)	59.6	60.6	96 (168)	62.2	57.1	112 (218)	63.2	51.4	-	-	-	-	-	-	-	-	-	-
SS-MD-SW	38 (69)	23.8	55.1	33 (71)	26.3	46.5	48 (86)	24.9	55.8	-	-	-	-	-	-	-	-	-	-
SS-LN-SW	27 (56)	19.3	48.2	14 (31)	11.5	45.2	23 (41)	11.9	56.1	-	-	-	-	-	-	-	-	-	-
SS-DE-CN-SW [b]	33 (59)	40.7	55.9	37 (72)	51.4	51.4	41 (81)	41.4	50.6	SS-AD-CN-SW	36 (61)	42.9	59.0	43 (82)	60.6	52.4	35 (75)	41.7	46.7
SS-DE-T-SW [b]	35 (61)	43.2	57.4	22 (40)	30.6	55.0	31 (57)	31.3	54.4	SS-AD-T-SW	11 (22)	13.1	50.0	14 (26)	19.7	53.8	23 (38)	27.4	60.5
SS-DE-W-SW [b]	13 (24)	16.0	54.2	13 (21)	18.1	61.9	27 (45)	27.3	60.0	SS-AD-W-SW	37 (63)	44.0	58.7	14 (29)	19.7	48.3	26 (49)	31.0	53.1
SS-DE-SH-SW d	50 (81)	56.3	61.7	9 (21)	61.7	42.9	60 (111)	60.7	54.1	SS-AD-SH-SW	50 (84)	57.5	59.5	50 (86)	62.8	58.1	52 (107)	66.0	48.6
SS-DE-CN-SH-SW [b]	19 (32)	38.0	59.4	21 (44)	45.7	47.7	19 (43)	31.7	44.2	SS-AD-CN-SH-SW	22 (35)	44.0	62.9	28 (50)	56.0	56.0	17 (50)	32.7	34.0
SS-DE-T-SH-SW [b]	19 (31)	38.0	61.3	14 (21)	30.4	66.7	21 (35)	35.0	60.0	SS-AD-T-SH-SW	5 (12)	10.0	41.7	9 (18)	18.0	50.0	15 (24)	28.8	62.5
SS-DE-W-SH-SW [b]	12 (18)	24.0	66.7	11 (17)	23.9	64.7	20 (33)	33.3	60.6	SS-AD-W-SH-SW	23 (37)	46.0	63.2	13 (18)	26.0	72.2	20 (33)	38.5	60.6
SS-DE-CN-SH-SW-SWFE	2	10.5	-	2	9.5	-	1	5.3	-	SS-AD-CN-SH-SW-SWFE	2	9.1	-	3	10.7	-	1	5.9	-
SS-DE-CN-SH-SW-SWUE	10	52.6	-	12	57.1	-	14	73.7	-	SS-AD-CN-SH-SW-SWUE	13	59.1	-	18	64.3	-	14	82.4	-
SS-DE-CN-SH-SW-SWW	7	36.8	-	7	33.3	-	4	21.1	-	SS-AD-CN-SH-SW-SWW	7	31.8	-	7	25.0	-	2	11.8	-
SS-DE-T-SH-SW-SWFE	4	21.1	-	2	14.3	-	1	4.8	-	SS-AD-T-SH-SW-SWFE	1	20.0	-	1	11.1	-	2	13.3	-
SS-DE-T-SH-SW-SWUE	13	68.4	-	7	50.0	-	16	76.2	-	SS-AD-T-SH-SW-SWUE	3	60.0	-	3	33.3	-	8	53.3	-
SS-DE-T-SH-SW-SWW	2	10.5	-	5	35.7	-	4	19.0	-	SS-AD-T-SH-SW-SWW	1	20.0	-	5	55.6	-	5	33.3	-
SS-DE-W-SH-SW-SWFE	1	8.3	-	3	27.3	-	1	5.0	-	SS-AD-W-SH-SW-SWFE	3	13.0	-	5	38.5	-	1	5.0	-
SS-DE-W-SH-SW-SWUE	6	50.0	-	3	27.3	-	17	85.0	-	SS-AD-W-SH-SW-SWUE	15	65.2	-	4	30.8	-	13	65.0	-
SS-DE-W-SH-SW-SWW	5	41.7	-	5	45.5	-	2	10.0	-	SS-AD-W-SH-SW-SWW	5	21.7	-	4	30.8	-	6	30.0	-
SS-DE-MD-SW [d]	16 (31)	21.5	51.6	17 (30)	22.6	56.7	28 (51)	27.9	54.9	SS-AD-MD-SW	22 (38)	26.0	57.9	16 (41)	29.9	39.0	20 (35)	21.6	57.1
SS-DE-CN-MD-SW [b]	7 (12)	43.8	58.3	9 (17)	52.9	52.9	16 (28)	57.1	57.1	SS-AD-CN-MD-SW	9 (17)	40.9	52.9	11 (25)	68.8	44.0	9 (12)	45.0	75.0
SS-DE-T-MD-SW [b]	8 (17)	50.0	47.1	6 (11)	35.3	54.5	8 (16)	28.6	50.0	SS-AD-T-MD-SW	4 (7)	18.2	57.1	5 (7)	31.3	71.4	5 (10)	25.0	50.0
SS-DE-W-MD-SW [b]	1 (2)	6.3	50.0	2 (2)	11.8	100.0	4 (7)	14.3	57.1	SS-AD-W-MD-SW	9 (14)	40.9	64.3	- (9)	0.0	-	6 (13)	30.0	46.2
SS-DE-CN-MD-SW-SWFE	2	28.6	-	1	11.1	-	2	12.5	-	SS-AD-CN-MD-SW-SWFE	2	22.2	-	6	54.5	-	1	11.1	-
SS-DE-CN-MD-SW-SWUE	2	28.6	-	5	55.6	-	7	43.8	-	SS-AD-CN-MD-SW-SWUE	3	33.3	-	3	27.3	-	7	77.8	-
SS-DE-CN-MD-SW-SWW	3	42.9	-	3	33.3	-	7	43.8	-	SS-AD-CN-MD-SW-SWW	4	44.4	-	2	18.2	-	1	11.1	-
SS-DE-T-MD-SW-SWFE	3	37.5	-	1	16.7	-	3	37.5	-	SS-AD-T-MD-SW-SWFE	1	25.0	-	2	40.0	-	2	40.0	-
SS-DE-T-MD-SW-SWUE	4	50.0	-	4	66.7	-	4	50.0	-	SS-AD-T-MD-SW-SWUE	2	50.0	-	1	20.0	-	2	40.0	-
SS-DE-T-MD-SW-SWW	1	12.5	-	1	16.7	-	1	12.5	-	SS-AD-T-MD-SW-SWW	1	25.0	-	2	40.0	-	1	20.0	-
SS-DE-W- MD -SW-SWFE	-	-	-	-	-	-	2	50.0	-	SS-AD-W- MD -SW-SWFE	3	33.3	-	-	-	-	2	33.3	-
SS-DE-W-MD-SW-SWUE	-	-	-	-	-	-	1	25.0	-	SS-AD-W-MD-SW-SWUE	2	22.2	-	-	-	-	1	16.7	-
SS-DE-W-MD-SW-SWW	1	100.0	-	2	100.0	-	1	25.0	-	SS-AD-W-MD-SW-SWW	4	44.4	-	-	-	-	3	50.0	-
SS-DE-LN-SW [d]	15 (32)	22.2	46.9	46 (82)	15.8	56.1	11 (21)	11.5	52.4	SS-AD-LN-SW	12 (24)	16.4	50.0	5 (10)	7.3	50.0	12 (20)	12.3	60.0
SS-DE-CN-LN-SW [b]	7 (15)	46.7	46.7	7 (11)	77.8	63.6	6 (10)	54.5	60.0	SS-AD-CN-LN-SW	5 (9)	41.7	55.6	4 (7)	80.0	57.1	9 (13)	75.0	69.2
SS-DE-T-LN-SW [b]	8 (13)	53.3	61.5	2 (8)	22.2	25.0	2 (6)	18.2	33.3	SS-AD-T-LN-SW	2 (3)	16.7	66.7	- (1)	0.0	-	3 (4)	25.0	75.0
SS-DE-W-LN-SW [b]	- (4)	-	0.0	- (2)	-	0.0	3 (5)	27.3	60.0	SS-AD-W-LN-SW	5 (12)	41.7	41.7	1 (2)	20.0	50.0	- (3)	-	0.0
SS-DE-CN-LN-SW-SWFE			-	3	42.9	-	1	16.7	-	SS-AD-CN-LN-SW-SWFE	-	-	-	-	-	-	2	22.2	-
SS-DE-CN-LN-SW-SWUE	5	71.4	-	1	14.3	-	5	83.3	-	SS-AD-CN-LN-SW-SWUE	4	80.0	-	2	50.0	-	5	55.6	-
SS-DE-CN-LN-SW-SWW	2	28.6	-	3	42.9	-	-	-	-	SS-AD-CN-LN-SW-SWW	1	20.0	-	2	50.0	-	2	22.2	-
SS-DE-T-LN-SW-SWFE	1	12.5	-	-	-	-	1	50.0	-	SS-AD-T-LN-SW-SWFE	1	50.0	-	-	-	-	1	33.3	-
SS-DE-T-LN-SW-SWUE	5	62.5	-	1	50.0	-	1	50.0	-	SS-AD-T-LN-SW-SWUE	1	50.0	-	-	-	-	2	66.7	-
SS-DE-T-LN-SW-SWW	2	25.0	-	1	50.0	-	-	-	-	SS-AD-T-LN-SW-SWW	-	-	-	-	-	-	-	-	-
SS-DE-W-LN-SW-SWFE	-	-	-	-	-	-	1	33.3	-	SS-AD-W-LN-SW-SWFE	-	-	-	1	100.0	-	-	-	-
SS-DE-W-LN-SW-SWUE	-	-	-	-	-	-	-	-	-	SS-AD-W-LN-SW-SWUE	1	20.0	-	-	-	-	-	-	-
SS-DE-W-LN-SW-SWW	-	-	-	-	-	-	2	66.7	-	SS-AD-W-LN-SW-SWW	4	80.0	-	-	-	-	-	-	-

Fr.= Frequency; Ef.= Effectiveness. a: The percentage refers to the relation FS/SS/DF about the total points analyzed. The effectiveness refers to the points won with second service by the player that serves; b: the percentage refers to the distribution of the points played with first service depending on the place (DE/AD); c: the frequency determines the number of points played with the pattern indicated (frequency of this pattern without considering the winner criteria –SW-). The percentage refers to the distribution of the points with first service depending on the zone (CN/T/W) and the effectiveness refers to the points won by the player that serves without considering the winning criteria (SW)
